# Vaginal mucosal vaccine for recurrent urinary tract infections

**Published:** 2007

**Authors:** Gagan Gautam, Rakesh Khera

**Affiliations:** Department of Urology and Renal Transplant, Fortis Flt. Lt. Rajan Dhall Hospital, Vasant Kunj, New Delhi - 110 070, India. E-mail: gagangg@gmail.com

## SUMMARY

The authors conducted a randomized double-blind placebo-controlled study to assess the clinical efficacy of vaginal mucosal immunization with a multivalent bacterial vaccine in women with recurrent urinary tract infections (UTIs). A total of 75 patients who had suffered three or more UTIs in the past one year, were randomly assigned to receive placebo only (n = 25), primary immunization without boosters (n = 24) or primary immunization plus boosters (n = 26) using vaginal suppositories containing placebo or vaccine. Vaccine suppositories contained 10 strains of heat-killed uropathogenic bacteria (a total of 10^9^ organisms from six strains of *E. coli* and one each of Proteus, *Morganella*, *Klebsiella* and *E. fecalis* in a polyethylene glycol base) and placebo suppositories had no vaccine organisms. Primary immunization consisted of three suppositories given at weekly intervals while boosters were given in three doses at monthly intervals after completion of the primary doses. All women were monitored for six months to record the number of infections and adverse events.

Analysis of data on UTIs caused by any bacteria showed the greatest difference in infection rates between patients in the vaccine plus boosters protocol compared to those receiving placebo only (*P* = 0.100). The proportion of patients remaining infection-free during the study was 16.7% in the placebo only arm, 25% in the vaccination without boosters arm and 46% in the vaccination with boosters arm. When only *E. coli* UTIs were considered in the analysis, UTI recurrence rates were significantly less in women given booster immunizations compared to placebo (*P* = 0.0015) and vaccination without boosters as compared to placebo (*P* = 0.038). Thirty per cent of patients in the placebo protocol, 57% in the vaccine without boosters protocol and 72.5% in the vaccine with boosters protocol remained free of *E. coli* infection.

When a subgroup analysis of sexually active women was performed, it was found that 72.7% (16/22) women receiving vaccine with boosters remained free of *E. coli* infection as compared to 17.6% (3/17) of those receiving placebo only (*P* = 0.0002). Furthermore, statistically significant lower *E. coli* infection rates were observed in vaccine-treated women with age < 52 years (*P* = 0.002), absence of a history of recurrent childhood UTIs (*P* = 0.003), greater than five UTIs in the previous year (*P* = 0.009), no history of a hysterectomy (*P* = 0.001), use of estrogen (*P* = 0.002) and oral contraceptive pills (*P* = 0.0001).

A quantitative analysis of anti *E. coli* IgA and IgG antibodies in the urine and vaginal fluid samples collected during the course of the study did not reveal any significant differences between the three groups. There were no significant adverse events associated with vaccine treatment. The authors concluded that vaginal mucosal immunization with a multivalent vaccine is safe and efficacious in reducing recurrence of *E. coli* UTIs with maximal benefit seen in sexually active women in the 20 to 50-year-old age group.

## COMMENTS

Urinary tract infections are a major cause of morbidity in women and *E. coli* is the predominant organism in both community-acquired and hospital-acquired forms of the disease. Long-term antibiotic prophylaxis is often instituted for patients with recurrent UTI not controlled by conservative measures. Although this is an effective intervention for controlling infections, it carries the disadvantage of antibiotic-related adverse effects and introduction of drug resistance, which can limit its use. Moreover, the chances of a recurrent UTI return to the baseline level as soon as the antibiotic is stopped.[1] As a result most of these unfortunate women are condemned to suffering from repeated bouts of infection, need frequent antibiotic treatments and remain prone to serious infections like acute pyelonephritis and its sequelae.

Recent studies on the induction of vaginal mucosal immunity to uropathogenic organisms through vaccine suppositories hold promise for the future to provide a convenient, effective and safe alternative to antibiotics in this group of patients.[2,3] The major advantage of a mucosal immunization is the theoretical induction of local immunity resulting in the production of IgA antibodies. This prevents the onset of infection directly at the level of the initial source. In this way, mucosal antibody formation via vaginal vaccination may be more effective than parenteral vaccination. Moreover, the use of a polyvalent whole cell vaccine would induce immunity against a variety of bacterial antigens, which can provide a wider range of protection against the whole gamut of uropathogenic bacteria.

However, there still remain some challenges to overcome before such a treatment can attain widespread clinical applicability. The adequate dosage and treatment schedule is still empirical and needs to be studied further. Improvement in terms of vaccine composition is required to achieve protection from non-*E. coli* -pathogens, which continue to cause large-scale morbidity and mortality in the hospital inpatient population. Moreover, further research needs to be done with regards to the mechanisms of protection and immunological basis for the success of this form of treatment.
